# FNIP1 Deficiency: Pathophysiology and Clinical Manifestations of a Rare Syndromic Primary Immunodeficiency

**DOI:** 10.3390/cimb47040290

**Published:** 2025-04-18

**Authors:** Samuele Roncareggi, Brian M. Iritani, Francesco Saettini

**Affiliations:** 1 Dipartimento Di Medicina e Chirurgia, Università Degli Studi Milano-Bicocca, 20900 Monza, Italy; s.roncareggi@campus.unimib.it; 2 The Department of Comparative Medicine, University of Washington, Seattle, WA 98195, USA; biritani@uw.edu; 3 Clinica Pediatrica, Fondazione IRCCS San Gerardo dei Tintori, 20900 Monza, Italy; 4 Centro Tettamanti, Fondazione IRCCS San Gerardo dei Tintori, 20900 Monza, Italy

**Keywords:** FNIP1 deficiency, inborn error of immunity, primary immunodeficiency, agammaglobulinemia, hypertrophic cardiomyopathy, neutropenia

## Abstract

Folliculin-interacting protein 1 (FNIP1) is a key regulator of cellular metabolism and immune homeostasis, integrating nutrient signaling with proteostasis. FNIP1 forms a complex with folliculin (FLCN) to regulate the mechanistic target of rapamycin complex 1 (mTORC1), functioning as a GTPase-activating protein (GAP) for RagC/D. Additionally, FNIP1 interacts with heat shock protein 90 (HSP90) and undergoes phosphorylation, glycosylation, and ubiquitination, which dynamically regulate its stability and function. Evidence from murine models suggests that FNIP1 loss disrupts immune cell development and mitochondrial homeostasis. However, FNIP1 deficiency in humans remains incompletely characterized, and its full phenotypic spectrum is likely underestimated. Notably, FNIP1-deficient patients exhibit immunological and hematological abnormalities, immune dysregulation, and metabolic perturbations, emphasizing its role in cellular adaptation to stress. Understanding the mechanistic basis of FNIP1 dysfunction in human tissues will be critical for delineating its contributions to immune and metabolic disorders and identifying targeted interventions.

## 1. Introduction

Over the last few years, the widespread application of large-scale genomic sequencing has led to the identification of novel germline variants associated with inborn errors of immunity (IEIs) [1]. These monogenic defects present a broad range of clinical phenotypes, often affecting both the immune and non-immune systems. Many IEIs are not limited to classical immunodeficiency but instead manifest as complex multisystemic syndromes, involving hematological, neurological, and metabolic abnormalities. This growing understanding of IEIs emphasizes the interplay between immune dysfunction and systemic disease [2,3,4,5].

Folliculin interacting protein 1 (FNIP1) deficiency was first identified in 2020 as a novel IEI, characterized by a broad spectrum of immune and non-immune manifestations [6,7]. The disorder is inherited in an autosomal recessive inheritance pattern, with pathogenic variants in FNIP1 leading to loss-of-function effects. FNIP1 deficiency is currently classified as a predominantly antibody deficiency, with patients exhibiting severe B-cell deficiency and hypogammaglobulinemia or agammaglobulinemia [8]. This deficiency results in a heightened susceptibility to recurrent infections, particularly bacterial infections affecting the respiratory tract. Neutropenia and monocytosis are frequent hematological findings.

Beyond the immune system, FNIP1 deficiency has been linked to a diverse array of non-immune abnormalities, suggesting a broader role for FNIP1 in human physiology. Notably, congenital heart defects have been described in all reported cases, suggesting a crucial function for FNIP1 in cardiac development and maintenance. Additionally, some patients exhibit neurological and muscular manifestations, including developmental delay, hypotonia, and structural brain abnormalities, further supporting the hypothesis that FNIP1 plays a role in multiple organ systems [6,7,9,10,11]. These findings reinforce the idea that FNIP1 deficiency is not solely an immunodeficiency but rather a syndromic disorder with systemic involvement.

Currently, only limited data are available in the literature characterizing the biological mechanisms and clinical outcomes of patients with biallelic *FNIP1* variants [6,7]. Most of our knowledge regarding FNIP1 function is derived from studies in animal and cellular models, which have demonstrated its essential role in cell signaling, development, maturation, and survival [12,13,14]. FNIP1 acts as a crucial adaptor protein, interacting with folliculin (FLCN) and AMP-activated protein kinase (AMPK) to regulate cellular metabolism, immune cell homeostasis, and key signaling pathways such as the mechanistic target of rapamycin complex 1 (mTORC1). Given its involvement in metabolic signaling, FNIP1 influences not only immune cell function but also broader physiological processes, including energy homeostasis and cellular proliferation in highly metabolic tissues.

The aim of this review is to summarize the molecular properties of FNIP1 and the association between germline FNIP1 mutations and immune-hematological and extra-hematological manifestations. We will highlight the possibility that the phenotypic spectrum of FNIP1 deficiency may be broader than currently recognized. Additionally, we will discuss the potential clinical implications of FNIP1 deficiency, including diagnostic challenges, treatment considerations, and future research directions, to provide guidance for clinicians and researchers working on this rare but fascinating disorder.

## 2. Structure and Function of FNIP1

**Figure 1:**
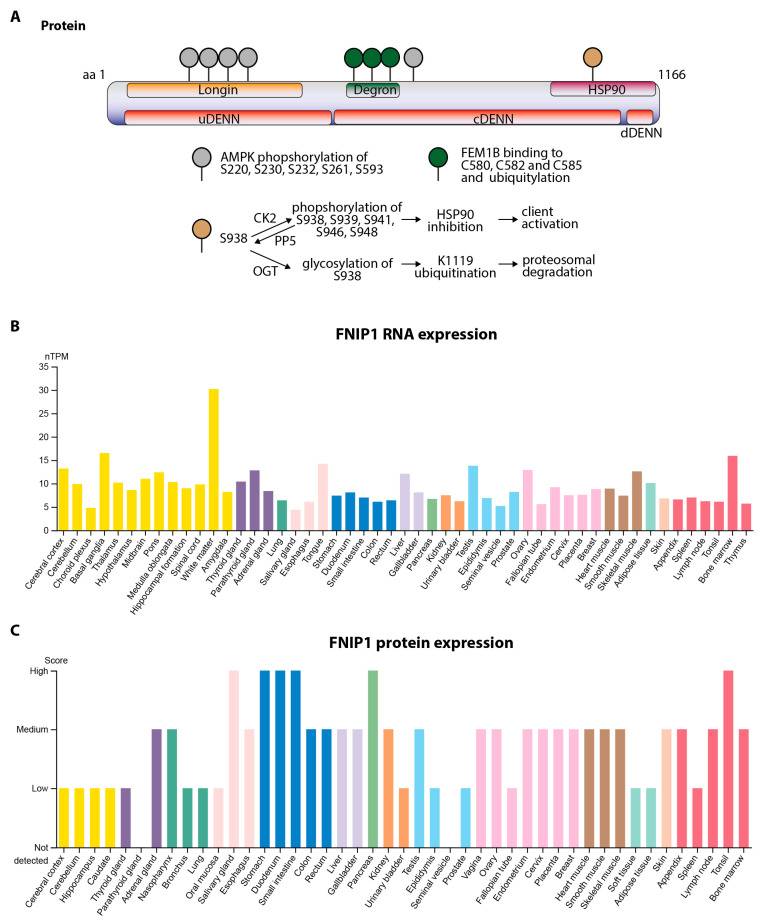
Protein domains, functional roles, and tissue-specific expression of FNIP1. (**A**) Schematic representation of FNIP1 protein structure and post-translational modifications. FNIP1 contains distinct domains, including longin, degron, and DENN-like domains. FNIP1 undergoes AMPK-dependent phosphorylation at serine residues S220, S230, S232, S261, and S593, regulating its function in metabolic sensing. Additional casein kinase 2 (CK2)-mediated phosphorylation at S938, S939, S941, S946, and S948 facilitates HSP90 inhibition and client protein activation. Conversely, protein phosphatase 5 (PP5)-mediated dephosphorylation allows for O-GlcNAc transferase (OGT)-dependent glycosylation of S938, thereby preventing HSP90 interaction and leading to K1119 ubiquitination and proteasomal degradation. The degron motif (C580, C582, and C585) enables FEM1B binding and FNIP1 ubiquitylation, facilitating degradation in response to reductive stress. (**B**) Tissue-specific FNIP1 RNA expression. Expression levels (nTPM) of FNIP1 transcripts across various human tissues. FNIP1 shows high expression in immune-related tissues, including the thymus, spleen, and bone marrow, as well as in metabolically active tissues such as the kidney and liver. (**C**) Tissue-specific FNIP1 protein expression. Protein expression levels, categorized as high, medium, low, or not detected with strong FNIP1 presence in immune and metabolic active tissues (https://v22.proteinatlas.org/ENSG00000217128-FNIP1/tissue, accessed on 4 March 2025).

The human *FNIP1* gene is located on chromosome 5 (5q31.1) and consists of 18 exons (GRCh38). The most well-characterized FNIP1 isoform encodes an 1166-amino-acid protein with a predicted molecular weight of approximately 130 kDa. FNIP1 is a highly conserved adaptor protein that plays a crucial role in cellular metabolism, immune regulation, and organelle homeostasis by integrating multiple signaling pathways. The FNIP1 protein structure comprises several domains that mediate its interaction and regulatory roles (Figure 1A).

### 2.1. Protein Domains, Post-Translational Modifications, Subcellular Localization, and Functional Roles of FNIP1

FNIP1 contains a longin domain, a structural motif involved in small GTPase regulation, trafficking, and vesicle formation. The longin domain is positioned upstream of the DENN (differentially expressed in normal and neoplastic cells) domain, which is common in multiple regulatory proteins, including folliculin (FLCN), FNIP1, FNIP2, NPR2-like (NPRL2), NPR3-like (NPRL3), C9orf72, and Smith–Magenis syndrome chromosomal region candidate gene 8 (SMCR8) [15,16]. These domains function in intracellular signaling, metabolic regulation, and immune responses (Figure 1A).

FNIP1 serves as a cytoplasmatic scaffolding protein, primarily interacting with FLCN to modulate the mechanistic target of the rapamycin complex 1 (mTORC1) pathway, which is a central regulator of lysosome and mitochondrial biogenesis, and autophagy, as well as AMPK, an enzyme that helps maintain energy and nutrient balance. These functions are particularly relevant in highly proliferative and energy-demanding cells, as nutrient utilization and mitochondrial metabolism are critical for cellular activation, survival, and immune function [17].

Under metabolic stress, FNIP1 serves as a critical link between AMPK and the stimulation of lysosome and mitochondrial biogenesis via TFEB. In response to low energy (low ATP/high AMP/ADP; Figure 2①), activated AMPK inhibits mTORC1-mediated energy and nutrient consumption and stimulates energy and nutrient production by enhancing mitochondrial and lysosomal biogenesis. Specifically, activated AMPK stimulates energy and nutrient production in part by phosphorylating FNIP1 at five conserved serine residues (S220, S230, S232, S261, and S593; Figure 1A and Figure 2②) [14]. FNIP1 recruits FLCN to lysosomes, where FLCN/FNIP1 acts as a GTPase-activating protein (GAP) complex promoting conversion of the GTPase RagC into the active GDP-bound state. The AMPK-driven phosphorylation of FNIP1 results in the inhibition of FLCN/FNIP1 GAP activity, the conversion of RagC into the inactive GTP-bound form, and the release of a complex of RagC and TFEB from the lysosome to the cytoplasm (Figure 2③). TFEB then translocates to the nucleus, where it induces the transcription of lysosomal and autophagy genes (Figure 2④). In addition, nuclear TFEB induces the expression of PGC1α, a master regulator of mitochondrial biogenesis. Thus, under conditions of energy deprivation and AMPK activation, FNIP1 serves as a key mediator controlling mitochondrial and lysosomal biogenesis, ensuring cellular adaptation to nutrient fluctuations and energy stress (Figure 2⑤⑥) [18].

**Figure 2:**
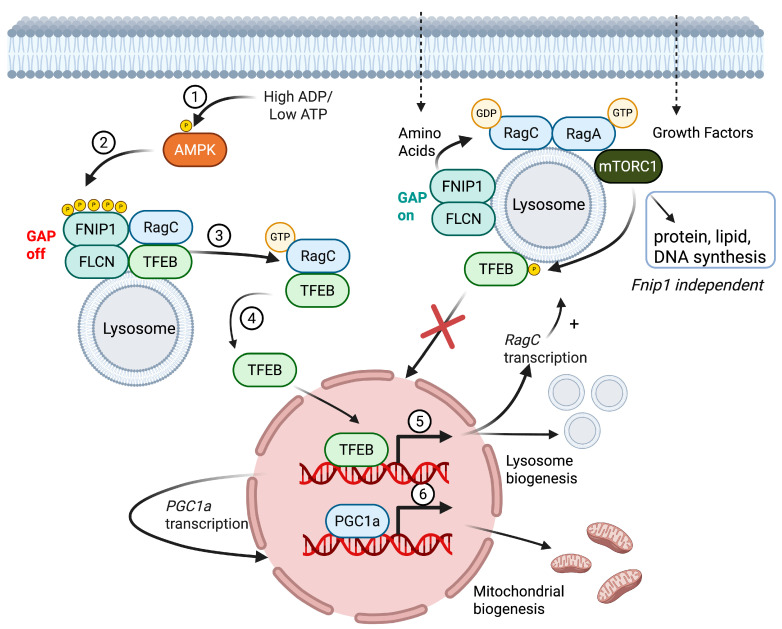
FNIP1 cellular signaling and metabolism. FNIP1, in complex with folliculin (FLCN), functions as a GAP (GTPase-activating protein) for RagC/D to regulate mTORC1 activity at the lysosome. Under low energy conditions (high AMP/low ATP) ①, FNIP1 is phosphorylated by AMPK, which prevents RagC activation ② and inhibits mTORC1 signaling. This results in the activation of TFE family transcription factors (TFEB and TFE3) ③④, which drive lysosomal ⑤ and mitochondrial biogenesis ⑥ via PGC1α and PGC1β transcription. Created in BioRender. Iritani, B. (2025), https://BioRender.com/f18p969, accessed on 4 March 2025.

In response to sufficient amino acids, mTORC1 is recruited to the lysosome membrane, where it stimulates protein, lipid, and purine synthesis needed for cellular growth and proliferation while inhibiting lysosome biogenesis and autophagy by phosphorylating and inhibiting the nuclear translocation of TFEB (transcription factor EB), a central transcriptional regulator of lysosome biogenesis [19]. FNIP1 modulates mTORC1 by recruiting FLCN to lysosomes, where FLCN/FNIP1 acts as a GAP complex promoting the conversion of the GTPase RagC into the active GDP-bound state (Figure 2②). This allows for the recruitment and activation of mTORC1. Both FNIP1 and its paralog, FNIP2, stabilize these interactions, ensuring the precise regulation of mitochondrial and lysosomal biogenesis (Figure 2) [14,20].

FNIP1 harbors a degron motif, which is crucial for cellular responses to reductive stress [21,22]. The degron motif consists of three invariant cysteine residues (C580, C582, and C585) that bind to FEM1B, an E3 ubiquitin ligase responsible for polyubiquitylating FNIP1. Under conditions of antioxidant signaling or mitochondrial inactivity, the oxidation state of the degron motif is reversed, facilitating FNIP1 degradation, and thereby restoring redox homeostasis and mitochondrial activity (Figure 1A) [21].

FNIP1 interacts with heat shock protein 90 (HSP90), a molecular chaperone essential for protein stabilization and folding, suggesting that FNIP1 plays a role in maintaining the integrity of its associated signaling complexes [23]. The phosphorylation of FNIP1 at S938 by casein-kinase-2 (CK2) initiates a phosphorylation cascade at S939, S941, S946, and S948, enhancing FNIP1’s interaction with HSP90. This interaction incrementally inhibits HSP90 ATPase activity, thereby modulating the activation of client proteins [24]. Additionally, FNIP1 contains multiple ubiquitination sites, implicating it in proteasomal degradation and cellular protein turnover, as reviewed by Zeng et al. [16]. The regulation of FNIP1 stability involves serine/threonine protein phosphatase 5 (PP5), which dephosphorylates FNIP1 at S938, allowing for the addition of O-GlcNAc (O-linked N-acetylglucosamine) to S938 by O-linked N-acetylglucosamine (GlcNAc) transferase (OGT). Glycosylation antagonizes FNIP1 phosphorylation, preventing its interaction with HSP90 and consequently promoting FNIP1 K1119 ubiquitination, leading to proteasomal degradation (Figure 1A) [24].

### 2.2. Transcript Variants and Tissue-Specific Expression

The functional significance of FNIP1 alternative transcript variants remains largely unexplored. The FNIP1 gene produces multiple transcript variants due to alternative splicing, which may contribute to tissue-specific regulation and functional specialization. These transcript variants may differentially influence FNIP1’s subcellular localization, stability, and post-translational modifications, potentially altering its function in a context-dependent manner.

Transcriptomic and proteomic data from the Human Protein Atlas (www.proteinatlas.com, accessed on 4 March 2025) [25,26] indicate that FNIP1 is widely expressed across human tissues, with differential mRNA and protein levels suggesting diverse physiological functions. Moderate to high FNIP1 mRNA expression is detected in hematopoietic and lymphoid organs, including the bone marrow, spleen, and lymph nodes. FNIP1 is expressed in immune cells, with significant levels in B-cells, T-cells, neutrophils, and monocytes, supporting its role in immune cell homeostasis (Figure 1B).

Beyond the immune system, FNIP1 mRNA and protein expression are detectable in non-immune tissues, including the kidneys, liver, lungs, cardiac and skeletal muscle, and brain, indicating broader physiological functions beyond immunity. Notably, FNIP1 mRNA is highly expressed in lung macrophages and brain oligodendrocytes. At the protein level, FNIP1 is most abundant in tonsils, lymph nodes, bone marrow, and the appendix, reinforcing its critical function in lymphoid tissues (Figure 1C).

However, FNIP1 expression at the RNA or protein level does not directly correlate with the observed clinical manifestations. Findings from Fnip1-deficient mouse models suggest that FNIP2 may partially compensate for the loss of FNIP1 in specific tissues, though this hypothesis has not yet been thoroughly investigated in humans.

### 2.3. Fnip1^−/−^ Models

In 2012, two research groups independently identified that disruptions in gene-encoding murine Fnip1 resulted in the absence of peripheral B-cells and agammaglobulinemia [12,13]. B-cell development in FNIP1-deficient mice was noted to be blocked at the intracellular immunoglobulin heavy chain (IgH)-positive/Ig light chain-negative (IgH^+^Igκ^−^) pre-B-cell stage. Enforced expression of IgM or Igκ transgenes failed to restore B-cell development, indicating the direct effect of Fnip1 loss on pre-B-cell signaling and survival during development [12,13]. Interestingly, Fnip1-deficient pre-B-cells also showed heightened sensitivity to apoptosis under nutrient-deprived conditions, and in vitro culture of Fnip1-deficient pre-B-cells in nutrient-rich media partially rescued the development of IgM+ B-cells [12]. These results were consistent with the existence of a “metabolic checkpoint” during B-cell development, where highly metabolic B-cells are tested to ensure that their metabolic capacity is sufficient.

Biochemical analysis of pre-B-cells from Fnip1-deficient mice has indicated either normal [13] or increased mTORC1 activation [12], increased AMPK activation [12,27], increased mitochondrial biogenesis [12,27], and enhanced autophagy [12,27]. These alterations correlate with the increased oxidative phosphorylation and glycolysis of cultured Fnip1-deficient pre-B-cells versus wildtype pre-B-cells [12]. Despite elevated mTORC1 activity, TFE3, a TFEB family member, is mostly nuclear in Fnip1-deficient pre-B-cells, which correlates with increased PGC1a expression and increased lysosome biogenesis and mitochondrial biogenesis [12,27]. These results support a model wherein activated AMPK mediates the deactivation of FNIP1, resulting in decreased FLCN/FNIP1 GAP activity, increased nuclear TFEB/TFE3, and increased lysosome and mitochondrial biogenesis.

Further analyses of Fnip1-deficient mice have revealed a multitude of other phenotypes, including defective invariant NKT cell development [28], hypertrophic cardiomyopathy [29], fiber type switch and altered mitochondrial dynamics in skeletal muscle [30], muscle-dysfunction-related bone loss [19], increased adipose tissue browning [31], and renal cyst formation [32]. Hypertrophic cardiomyopathy in FNIP1-deficient mice is associated with increased left ventricular (LV) mass. Electrocardiography analyses have revealed a shortened PR interval and a prolonged QRS complex, consistent with ventricular pre-excitation. Histological analyses have shown increased glycogen accumulation and pulmonary and hepatic congestion [29].

One of the most dramatic phenotypes in Fnip1-deficient mice has been observed in skeletal muscle. *Fnip1*^−/−^ skeletal muscle is noted to be significantly deeper red in coloration relative to control mice, due in part to a shift in the skeletal muscle fiber type toward fibers with increased type I fiber characteristics, including increased levels of myoglobin, cytochrome C, MyH7, and troponin, which is associated with reduced contraction fatigue [30]. Mitochondria density dramatically increases, which correlates with increased AMPK activation, increased PGC1a levels, and increased oxidative metabolism [30]. Activated AMPK has been found to control mitochondrial function, electron transport chain complex assembly, and muscle fuel utilization via the phosphorylation of Fnip1 at serine220 [20]. Capillary density is also significantly increased in Fnip1-deficient skeletal muscle [30], which has been reported to be associated with increased macrophage recruitment [33].

Adipose-specific disruption of Fnip1 has been found to control adipose tissue browning and glucose homeostasis due to increased UCP1 levels and increased mitochondria levels, which support augmented mitochondrial respiration [31]. Mechanistically, the loss of FNIP1 results in increased intracellular Ca^2+^ levels and the activation of a Ca^2+^-dependent thermogenic program in adipocytes. Interestingly, the adipose-specific loss of FLCN also results in adipose tissue browning [34]. The loss of FLCN results in the chronic hyperactivation of AMPK; increased UCP1; increased PGC1α; and increased estrogen-related receptor a (ERRa), which promotes mitochondria biogenesis.

Loss-of-function mutations in the *BHD* gene encoding FLCN are known to result in Birt–Hogg–Dubé syndrome (BHDS) in humans, a rare disease characterized by benign skin tumors (fibrofolliculomas), lung cysts, and renal cancers [35]. Whereas the homozygous deletion of *BHD* in mice is embryonic lethal, heterozygous *BHD* mice survive and develop renal cysts and solid tumors with age, thus mimicking humans with BHDS [36]. Although Fnip1-null mice do not develop renal tumors, small renal cysts develop consistently and are characterized by increased mTORC1 activation and alterations in the expression of genes associated with PKD in humans, including amino acid and ion transporters, cell adhesion, and inflammation [32]. Interestingly, although the targeted deletion of Fnip2 in mice does not result in observable phenotypes, kidney-specific disruption of both Fnip1 and Fnip2 leads to severe PKD, which eventually progresses to cancer [37]. These results suggest that in some tissues, Fnip1 and Fnip2 can compensate for the absence of the other member and that the phenotypes of Fnip1/2 loss largely recapitulate the absence of FLCN.

One of the major conundrums is why the loss of Fnip1 and Flcn often results in the hyperactivation of mTORC1 in vivo, whereas biochemical studies in cell lines suggest that the complex is important for activating mTORC1 at the lysosomal membrane [17,37,38]. Napolitano et al. found that the disruption of FLCN inhibited the abilities of mTORC1 to phosphorylate and inhibit TFEB, leading to increased nuclear TFEB, lysosomal biogenesis, and increased mitochondrial biogenesis [39]. However, the loss of FLCN did not inhibit the abilities of mTORC1 to activate protein, lipid, and DNA synthesis. In addition, increased nuclear TFEB led to the transcriptional induction of RagC/D, leading to the forward feedback activation of mTORC1-mediated cell growth. Hence, the FLCN/FNIP complex appears to be important for the mTORC1-driven inhibition of lysosome and mitochondrial biogenesis but not mTORC1-driven cell growth. Loss-of-function mutations in FLCN or FNIP1 lead to increased TFEB-driven lysosome and mitochondrial biogenesis, as well as forward-feedback-driven mTORC1 activation and cell growth.

## 3. Clinical Features of FNIP1 Deficiency

### 3.1. Germline FNIP1 Mutations and Disease

FNIP1 deficiency is caused by homozygous or compound-heterozygous loss-of-function mutations in the *FNIP1* gene. Identified variants include missense, nonsense, splice-site, and frameshift mutations, leading to a complete loss of FNIP1 protein function [6,7,9,10,11]. In some cases, complex genetic mechanisms such as uniparental disomy or copy-number variations have been implicated, expanding the spectrum of genetic alterations associated with this condition. Protein expression was tested in T lymphocytes in six patients, resulting in absent protein expression in all cases [6,7,9]. In one individual, FNIP1 mRNA in peripheral blood mononuclear cells was determined, resulting in significantly decreased but not absent FNIP1 mRNA, most likely due to incomplete nonsense-mediated decay [7].

**Figure 3:**
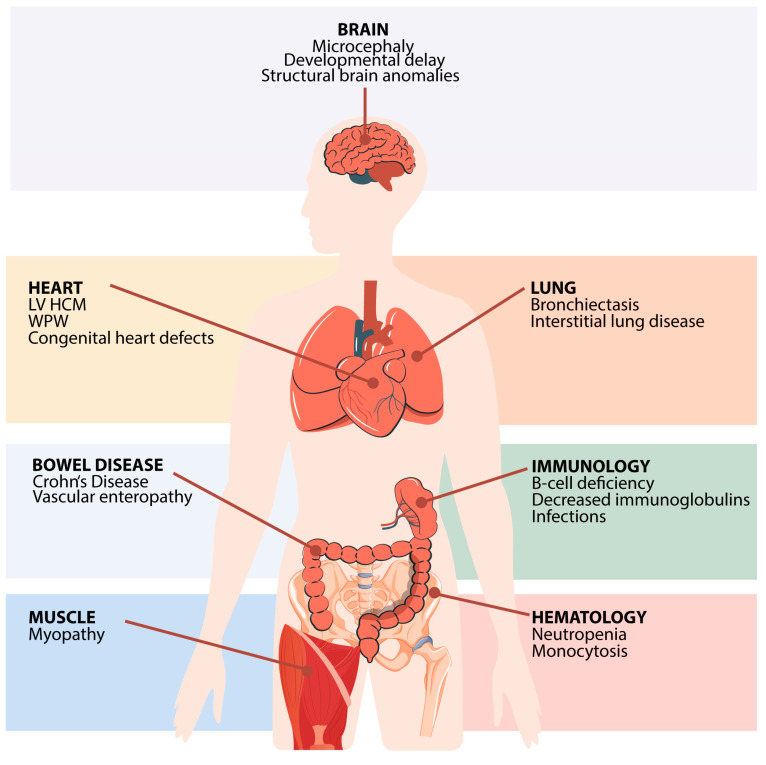
Clinical features of FNIP1 deficiency by organ system. Common clinical findings are shown by the affected organ system. Vectors from Freepik. LV HCM = left ventricular hypertrophic cardiomyopathy. WPW = Wolff–Parkinson–White syndrome.

To date, nine patients with biallelic *FNIP1* mutations have been reported across five studies [6,7,9,10,11], with one additional sibling presenting with a consistent phenotype but passing away prior to genetic testing [11]. Consanguinity was present in 5 out of 10 cases, supporting an autosomal recessive inheritance pattern [6,7,10]. FNIP1 deficiency presents with a broad spectrum of clinical manifestations, affecting both the immune and non-immune systems (Figure 3 and Table 1). Most affected individuals are children (<14 years old), with only two described adult cases (25 and 30 years old) [7,9]. Given the small cohort and the short available follow-up, some manifestations may not yet have fully emerged.

### 3.2. Hematological and Immunological Findings

FNIP1 deficiency is characterized by profound B-cell lymphopenia and antibody deficiency, often resulting in hypogammaglobulinemia or agammaglobulinemia.

Bone marrow flow cytometry in two cases revealed early B-cell differentiation defects, with either pro-B-pre-B arrest [6] or an increase in earlier maturation stages (pro-B and pre-B1), coupled with a significant reduction in immature B-cells [7]. Bone marrow B-cell progenitors showed the constitutive hyperactivation of mTORC1 downstream targets, including p4EBP1 and pAkt473, underscoring FNIP1’s role in early B-cell development [7]. Peripheral blood lymphocyte subset analysis consistently demonstrated severe B-cell depletion in eight of nine patients, with six individuals displaying profoundly low B-cell counts (<2% of lymphocytes) [6,7,9,10,11]. When the B-cell absolute number is sufficient to allow for the investigation of B-cell subsets, transitional B-cells are either normal or decreased, while switched memory B-cells are undetectable or reduced, thus reinforcing FNIP1’s critical role in early B-cell maturation. FNIP1’s role in human B-cell mitochondria has been shown in FNIP1-deficient circulating B-cells, which exhibited increased mitochondrial numbers and membrane potential compared to healthy controls [7]. Among three individuals with available data, vaccine responses were absent or decreased [6,7]. B-cell proliferation to antigens or mitogens has not been assessed. Immunoglobulin profiling confirms a profound humoral defect, with 9/10 patients exhibiting reduced serum immunoglobulin levels. Specifically, serum IgG and IgM levels were decreased in 9/10 individuals, while IgA deficiency was noted in 8/10 cases [6,7,9,10,11]. Data regarding autoantibodies are not available.

T-cell abnormalities are also notable, with increased absolute counts of CD3+, CD4+, and CD8+ reported in 6/9, 5/9, and 7/9 patients, respectively [6,7,9,10,11]. Standard lymphocyte proliferative responses to specific antigens and mitogens are usually normal. However, prolonged stimulation (up to 11 days), resulted in enhanced CD8+ apoptosis in one individual [7]. Natural killer (NK) cell counts are generally normal (seven of eight cases) [6,7,10,11].

Complete blood count (CBC) abnormalities are common. White blood cell abnormalities have been reported in three out of nine patients, with two individuals showing leukopenia [7,10] and one having leukocytosis [7]. Neutropenia (either chronic and severe or intermittent) is a predominant feature, present in 6 out of 10 patients [6,7,9,10], highlighting a defect in the myeloid compartment. Monocyte abnormalities have been detected, with three of six patients exhibiting monocytosis [5,6] and one presenting with monocytopenia [5]. However, in contrast to known severe congenital neutropenia, a bone marrow examination of one patient revealed no promyelocyte-myelocyte arrest [6]. Moreover, clonal evolution has not been observed in any patients.

### 3.3. Heart Disease

Cardiac anomalies are a hallmark of FNIP1 deficiency, with all the reported patients having heart disease. The most consistent finding is hypertrophic cardiomyopathy, documented in 10/10 patients [6,7,9,10,11], affecting the left ventricle in 9/10 and both ventricles in 1 patient (Spivak). Several individuals developed Wolff–Parkinson–White (WPW) syndrome, a pre-excitation syndrome that predisposes individuals to tachyarrhythmias, suggesting a direct role for FNIP1 in cardiac electrical conduction pathways [6,7,9,10]. FNIP1 deficiency may be a previously unrecognized cause of sudden unexplained death in affected individuals. Additionally, congenital heart defects, including interatrial communications and mitral/tricuspid valve regurgitation, have been consistently observed [7,10,11]. Cardiac MRI was performed on two patients, with one case specifically assessing myocardial glycogen accumulation [7]. Unlike *Fnip1^−/−^* murine models [33], glycogen accumulation was not detected.

### 3.4. Infectious Susceptibility

Recurrent infections are a universal finding in this cohort, affecting 9 of 10 patients from infancy [6,7,9,10,11]. Most individuals experience early-onset infections (<1 year of age). Infections primarily target the respiratory, gastrointestinal, and skin/mucosal barriers, often requiring hospitalization and prolonged antimicrobial therapy.

Respiratory tract infections are among the earliest and most frequent complications, with recurrent upper and lower respiratory infections documented in nearly all patients [6,7,9]. These infections involve both viral and bacterial pathogens, including encapsulated bacteria such as *Streptococcus pneumoniae* and *Haemophilus influenzae*. Pneumonia, otitis media, laryngitis, and bacterial meningitis are reported alongside opportunistic bacterial infections, with cases of *Pseudomonas aeruginosa* and *Acinetobacter haemolyticus* septicemia.

Gastrointestinal infections are also common [6,7,11], with severe gastroenteritis caused by Rotavirus or Campylobacter, sometimes leading to hypovolemic shock. Salmonella infections, including non-typhi Salmonella, have been isolated in stool cultures, further suggesting gastrointestinal immune dysregulation. Persistent diarrhea requiring hospitalization has been observed, contributing to failure to thrive.

Mucocutaneous infections are also frequent [7,9,11], including recurrent gingivitis, skin abscesses, and cellulitis, reflecting a broad susceptibility to both common and opportunistic infections, underscoring the critical role of FNIP1 in immune homeostasis.

### 3.5. Pulmonary Involvement

Recurrent and severe sinopulmonary infections start within the first year of life. Chronic lung disease was diagnosed in five of eight assessed individuals (in two cases, lung imaging was not mentioned in the published papers) [6,7], indicating a high prevalence of pulmonary complications with onset at childhood age. Bronchiectasis occurred in three out of eight cases, with one case requiring lobectomy [6,7]. Additionally, fibrosis and interstitial lung disease were reported in two patients [6].

The frequent overlap between chronic infections and early-onset and progressive pulmonary disease suggests an underlying defect in both humoral immunity and lung-specific immune defenses. Given the profound B-cell lymphopenia and neutropenia, it remains challenging to delineate the precise contribution of these immunodeficiency defects from potential dysfunctions in other cell types affected by FNIP1 deficiency. Notably, FNIP1 is highly expressed in lung macrophages, club cells, fibroblasts, and other epithelial cells in the lungs [25,26]. These cells play key roles in innate immune defense, surfactant regulation, and tissue repair. The loss of FNIP1 may compromise their ability to respond appropriately to environmental stimuli or pathogens, leading to chronic inflammation and/or the impaired clearance of pathogens. FNIP1 deficiency may contribute to the onset of chronic lung disease secondary to recurrent infections through multiple interconnected mechanisms involving immune dysregulation, mitochondrial dysfunction, and impaired cellular stress responses, particularly in lung-resident cells.

### 3.6. Muscular Abnormalities

The role of FNIP1 in skeletal muscles has been demonstrated in murine models [12,30]. In humans, muscular involvement was reported in three patients, all presenting with motor developmental delay affecting both gross and fine motor skills [6]. Clinical findings included lower limb hypotonia with a broad-based gait and gluteal/thigh muscle hypertrophy. A muscular biopsy was performed on one individual (P2), revealing mitochondria with atypical structure and surrounded by increased numbers of lipid droplets, consistent with metabolic myopathy [6]. While these findings suggest a primary mitochondrial dysfunction, routine muscle biopsies were not conducted on all patients, making it difficult to distinguish between myopathic versus neurological origins of muscle weakness and hypotonia.

Hypotonia was reported in one additional patient (II. 1) [11], while another patient with global developmental delay had locomotor function below age-matched normal ranges [7], suggesting that muscular involvement may be underreported or overlooked.

### 3.7. Central Nervous System (CNS) and Dysmorphic Features

Neurological manifestations are variable but present in a subset of patients. Developmental or cognitive delay was reported in 6 of 10 cases [6,7,9,10,11], suggesting that FNIP1 plays a role in neurodevelopment. While the exact mechanism remains unclear, the expression of the FNIP1 protein in the central nervous system supports its potential involvement in brain development and function.

Two patients also exhibited altered gastrointestinal motility or postnatal uninterruptable myoclonia [6], further implicating neuromuscular dysfunction.

Microcephaly was documented in four of nine individuals [6,7,10], while brain abnormalities were identified in two of three patients who underwent imaging studies [7,10,11]. The reporting findings included delayed myelinization, wider ventricles and sulci, a thin corpus callosum, and hypoplasia of the posterior cerebellum.

Dysmorphic features are infrequently reported but have been documented in at least two patients. Craniofacial abnormalities (hypertelorism, a low-set ear line, and a high palate), suggesting a possible syndromic phenotype, were reported in one case [10]. Another patient was described as having dysmorphic features [11], though specific details were not provided. Further studies are needed to determine whether FNIP1 deficiency is consistently associated with a characteristic dysmorphic presentation.

### 3.8. Gastrointestinal and Renal Manifestations

Gastrointestinal involvement was observed in 4 of 10 patients [6,7,10,11]. One individual was diagnosed with inflammatory bowel disease (IBD) that required multiple bowel surgeries [7]. Another patient, who also had chronic neutropenia, exhibited vascular enteropathy and recurrent perianal abscesses [9], raising concerns about underlying IBD. One patient exhibited chronic diarrhea that only partially improved after immunoglobulin replacement therapy was started [11]. Although data regarding the presence of autoantibodies was not available, large cohorts of patients with agammaglobulinemia reported that a significant number of patients (up to 28%) may present with autoimmune/autoinflammatory complications (arthritis, inflammatory bowel disease, or other inflammatory conditions) [40,41]. Gastrointestinal motility alterations were noted in another case, suggesting a broader enteric involvement beyond immune deficiency [6]. Given that FNIP1 is expressed in the gastrointestinal tract, its deficiency may contribute to mucosal immune dysregulation, although other mechanisms may also be implicated in disease pathogenesis.

Unlike many IEIs, renal involvement was notably absent in this cohort, despite FNIP1-deficient murine models exhibiting kidney abnormalities. However, renal involvement has not been systematically investigated, and given the limited sample size, kidney function in FNIP1-deficient individuals warrants further study, leaving the possibility that renal manifestations may emerge over time.

### 3.9. Treatment and Clinical Outcomes

Treatment strategies focus primarily on immunoglobulin replacement therapy (IgRT), which was administered to 9 of 10 patients [6,7,9,10,11]. This approach successfully reduced infection frequency but did not seem to prevent the early onset of complications, particularly regarding pulmonary complications. Low-dose granulocyte colony-stimulating factor (G-CSF) was used in 2 of 10 patients with severe neutropenia and resulted in the prompt improvement of the absolute neutrophil count [7,9], though its long-term efficacy and safety in terms of clonal evolution remains unclear. One patient received prophylactic antibiotics to prevent bacterial infections [7], and another required supplemental oxygen for respiratory insufficiency [7].

At the time of publication, 6 of 10 patients were alive, while 4 had died. Reported causes of death included severe infections (two cases) [7,9], cardiogenic shock [11], and unexplained sudden death [6]. The progressive nature of left ventricular hypertrophic cardiomyopathy (LV HCM) and the occurrence of fatal arrhythmias remain major concerns, necessitating close cardiac monitoring in all FNIP1-deficient individuals.

Early diagnosis and the timely initiation of IgRT are critical for preventing severe infections and improving quality of life. However, given the multisystemic involvement, FNIP1-deficient patients require multidisciplinary management, including pulmonary, cardiac, and neuromuscular evaluations, to anticipate and mitigate disease-related complications.

## 4. Conclusions

Studies in murine models have provided crucial insights into the role of FNIP1 in immune cell development, metabolism, and organ function. FNIP1-deficient mice exhibit profound B-cell maturation defects, metabolic abnormalities, and cardiomyopathy, mirroring many of the clinical manifestations observed in human FNIP1 deficiency. However, significant gaps remain in our understanding of FNIP1 function in human tissues, particularly in non-hematopoietic compartments such as the lungs, muscles, and nervous system.

Given the rarity of FNIP1 deficiency, the full phenotypic spectrum has likely not been fully captured, and many affected individuals may remain undiagnosed. IEIs are frequently associated with an increased risk of malignancy [42]. Several immune compartments, including B and myeloid lineages that are affected by FNIP1 deficiency, have been implicated in tumor predisposition in different IEIs. Despite these general associations, tumor development has not been reported in patients with FNIP1 deficiency to date. Similarly, FNIP1-deficient mice do not develop spontaneous tumors, further suggesting that FNIP1 loss alone does not inherently promote malignant transformation in hematopoietic or non-hematopoietic tissues. With reported cases being in childhood or early adulthood, the variable presentation and multisystemic involvement suggest that additional manifestations, including potential late-onset complications, could emerge over time. Comprehensive genetic screening and functional studies are needed to better define genotype–phenotype correlations and improve diagnostic rates.

FNIP1 is a key regulator of metabolic and signaling pathways, including mTOR and AMPK, which are already well-established drug targets. The pharmacological modulation of these pathways could represent a potential therapeutic strategy, particularly for managing metabolic and cardiac complications. mTOR inhibitors or metabolic modulators may provide targeted approaches to mitigating disease progression. Additionally, given the profound immune defects associated with FNIP1 deficiency, hematopoietic stem cell transplantation (HSCT) could be considered in severe cases, particularly in those with life-threatening infections or progressive immunodeficiency. However, the risks and benefits of HSCT in this context remain unclear and require further investigation.

Moving forward, expanding patient registries, leveraging multi-omics approaches, and utilizing patient-derived cellular models will be essential to elucidating the full biological and clinical implications of FNIP1 deficiency and identifying viable therapeutic interventions.

## Figures and Tables

**Table 1:** Main manifestations in the reported patients.

	Summary	Niehues, 2020 [6]	Saettini, 2021 [7]	Moreno-Corona, 2023 [9]	Ulaş, 2024 [10]	Spivak, 2025 [11]
**Number of patients**	**10**	3	3	1	1	2
**Study design**		WES, *n* = 1 Sanger sequencing, *n* = 2	WES, *n* = 3	WES = 1	WES = 1	WES = 1
**Consanguinity**	**5/10**	3/3	1/3	0/1	1/1	0/2 **
**Age at the time of the description**		P1: 7 months P2: 14 years P3: 8 months	P1: 3 years P2: 9 years P3: 25 years	30 years	11 months	II. 4: 1 year II. 1: 2 months
**Complete Blood Count**						
**Anemia**	**3/4**	NA	2/3	NA	1/1	NA
**White blood cells**						
**Leukocytosis**	**1/9**	0/3	1/3	NA	0/1	0/2
**Leukopenia**	**2/9**	0/3	1/3	NA	1/1	0/2
**Neutropenia**	**6/10**	2/3	2/3	1/1	1/1	0/2
**ALC, normal**	**5/9**	0/3	3/3	NA	0/1	2/2
**ALC, increased**	**2/9**	2/3	0/3	NA	0/1	0/2
**Monocytosis**	**3/6**	1/3	2/3	NA	NA	NA
**Monocytopenia**	**1/6**	1/3	0/3	NA	NA	NA
**Platelets, normal**	**3/3**	NA	3/3	NA	NA	NA
**Lymphocyte Subsets**						
**T-cells**						
**Increased CD3+**	**6/9**	3/3	2/3	1/1	0/1 *	0/1
**Increased CD4+**	**5/9**	3/3	2/3	0/1	0/1 *	1/1
**Increased CD8+**	**7/9**	3/3	2/3	1/1	0/1 *	1/1
**B-cells**						
**Decreased B-cells**	**8/9**	2/3	3/3	1/1	1/1	1/1
**B-cells < 2%**	**6/9**	0/3	3/3	1/1	1/1	1/1
**NK cells, normal**	**7/8**	3/3	3/3	NA	0/1 *	1/1 *
**Decreased NKT cells**	**1/3**	NA	1/3	NA	NA	NA
**Immunoglobulin**						
**IgG**	**9/10**	2/3	3/3	1/1	1/1	2/2
**IgA**	**8/10**	1/3	3/3	1/1	1/1	2/2
**IgM**	**9/10**	2/3	3/3	1/1	1/1	2/2
**Vaccine responses, low/absent**	**3/3**	2/2	1/1	NA	NA	NA
**T-cell proliferation, defective**	**1/6**	0/3	1/3	NA	NA	NA
**Bone Marrow**						
**BM B-cells**	**2/2**	1/1 B-cell maturation defect at the pre-B-cell stage with an increase in CD34^−^CD10^+^ CD21^low^-B-cell precursors	One out of one, no-maturation block; increase in earlier maturation stages (pro-B and pre-B1) with a significant reduction in immature B-cells.	NA	NA	NA
**BM myeloid**	**1/1**	NA	One out of one, delayed granulocyte maturation; no overt arrest.	NA	NA	NA
**Infections**	**9/10**	3/3	3/3	1/1	1/1	1/2
**Onset < 1 year**	**9/10**	3/3	3/3	1/1	1/1	1/2
**Heart disease**	**10/10**	3/3	3/3	1/1	1/1	2/2
**LV HCM**	**10/10**	3/3	3/3	1/1	1/1	2/2
**WPW**	**5/10**	2/3	1/3	1/1	1/1	0/2
**Other ^+^**	**4/10**	0/3	1/3	0/1	1/1	2/2
**Chronic Lung disease**	**5/8**	2/3	3/3	NA	NA	0/2
**CNS**						
**Microcephaly**	**4/10**	2/3	1/3	0/1	1/1	0/2
**Developmental or cognitive delay**	**6/10**	2/3	1/3	1/1	1/1	1/2
**Brain abnormalities**	**2/3**	NA	1/1	NA	1/1	0/1
**Muscle**	**4/8**	3/3	0/3	NA	NA	1/2
**Bowel disease**	**4/10**	1/3	1/3	1/1	0/1	1/2
**IBD**	**1/10**	0/3	1/3	0/1	0/1	0/2
**Other ^++^**	**3/10**	1/3	0/3	1/1	0/1	1/2
**Dysmorphic features**	**2/10**	0/3	0/3	NA	1/1	1/2
**Kidney**	**0/9**	0/3	0/3	NA	0/1	0/2
**Treatment**						
**IgRT**	**9/10**	3/3	3/3	1/1	1/1	1/1
**G-CSF**	**2/10**	0/3	1/3	1/1	0/1	0/2
**Antibiotic prophylaxis**	**1/10**	0/3	1/3	0/1	0/1	0/2
**Oxygen**	**1/10**	0/3	1/3	0/1	0/1	0/2
**Status at the time of the description, alive**	**6/10**	2/3	2/3	0/1	1/1	1/2
**Cause of death**		P2: sudden death	P2: pneumonia	Heart failure associated with SARS-CoV-2 infection		II. 1: cardiogenic shock

NA = not available. * = percentages. ** = sibling of the proband, with similar clinical and laboratory findings, succumbed prior to genetic testing. ^+^ = congenital heart defects, including interatrial communications and mitral/tricuspid valve regurgitation. ^++^ = altered gastrointestinal motility, vascular enteropathy, or chronic diarrhea.
